# Epidemiologic Characteristics Determining the Choice of Direct-Acting Antiviral Therapy in HCV Patients: An Italian Real-World Evidence Study

**DOI:** 10.3390/pathogens14111177

**Published:** 2025-11-18

**Authors:** Nicola Pugliese, Fabio Conti, Valerio Rosato, Paolo Gallo, Stefano Gitto, Marco Riglietta, Francesca Frigerio, Valentina Perrone, Chiara Veronesi, Maria Cappuccilli, Luca Degli Esposti, Alessandra Mangia, Loreta A. Kondili

**Affiliations:** 1 Department of Biomedical Sciences, Humanitas University, 20072 Pieve Emanuele, MI, Italy; nicola.pugliese@humanitas.it; 2 Division of Internal Medicine and Hepatology, Department of Gastroenterology, IRCCS Humanitas Research Hospital, 20089 Rozzano, MI, Italy; 3 Dipartimento di Medicina Interna, Ospedale degli Infermi di Faenza, 48018 Faenza, FC, Italy; fabio.conti@auslromagna.it; 4 Unità di Medicina Interna ed Epatologia, Ospedale Evangelico Villa Betania, 80147 Napoli, NA, Italy; valeriorosato@gmail.com; 5 UOS Medicina Clinica ed Epatologia, Policlinico Universitario Campus Bio-Medico, 00128 Roma, RM, Italy; paolo.gallo@policlinicocampus.it; 6 Department of Experimental and Clinical Medicine, University of Florence, 50141 Firenze, FI, Italy; 7 UOC Dipendenze, ASST Papa Giovanni XXIII, 24127 Bergamo, BG, Italy; mriglietta@asst-pg23.it; 8 Gilead Sciences, 20146 Milano, MI, Italy; francesca.frigerio@gilead.com; 9 CliCon S.r.l. Società Benefit, Health, Economics & Outcomes Research, 40137 Bologna, BO, Italy; chiara.veronesi@clicon.it (C.V.); maria.cappuccilli@clicon.it (M.C.); luca.degliesposti@clicon.it (L.D.E.); 10 UOS Epatologia, Istituto di Ricovero e Cura (IRCCS) “Casa Sollievo della Sofferenza”, 71013 San Giovanni Rotondo, FG, Italy; a.mangia@tin.it; 11 Center for Global Health, Istituto Superiore di Sanità, 00161 Rome, RM, Italy; loreta.kondili@iss.it; 12 Internal Medicine, UniCamillus International Medical Universityof Health Sciences, 00131 Rome, RM, Italy

**Keywords:** drug–drug interactions (DDIs), glecaprevir/pibrentasvir, hepatitis C virus (HCV), pangenotypic direct-acting antivirals, sofosbuvir/velpatasvir

## Abstract

Pangenotypic direct-acting antivirals (pDAAs) have transformed hepatitis C virus (HCV) treatment. In Italy, sofosbuvir/velpatasvir (SOF/VEL) and glecaprevir/pibrentasvir (GLE/PIB) are available. While both show similar efficacy, differences in patient profiles and potential drug–drug interactions (DDIs) may influence treatment choice. This study examined factors affecting pDAA selection and potential prescribing gaps. Using administrative databases (2018–2023) covering 3.7 million citizens, HCV patients were divided into SOF/VEL and GLE/PIB cohorts and compared by demographic, clinical, and therapeutic data. Among 5565 patients, 2837 (51%) received SOF/VEL and 2728 (49%) received GLE/PIB. SOF/VEL patients were older (60.8 vs. 57.6 years, *p* < 0.001) and had more comorbidities: diabetes (24% vs. 17%), mental disorders (22% vs. 14%), cancer (14% vs. 9%), and cardiovascular disease (31% vs. 22%). Hospitalization rates were higher (19% vs. 13%), as were exemption codes for chronic hepatitis (58% vs. 50%) and hypertension (32% vs. 23%). Polypharmacy was more common with SOF/VEL; 25% used ≥10 non-pDAA drugs (vs. 17%), and mean medications per patient were higher (6.3 ± 5.6 vs. 4.9 ± 5.2). SOF/VEL was often used for older, frailer patients, likely due to a more favourable DDI profile. These prescribing trends highlight the importance of tailoring pDAA choice to patient comorbidity profiles, ensuring appropriate and individualized HCV treatment.

## 1. Introduction

The hepatitis C virus (HCV) infection is a major cause of liver disease worldwide, with significant morbidity and mortality. For the year 2019, the World Health Organization (WHO) estimated around 58 million people with chronic HCV infection and 399,000 deaths [1]. The burden of HCV has been changing over the years in historically high-endemic areas including Italy. Recent Italian studies have documented a shifting epidemiology in the general population compared to the past. Demographic transitions, the adoption of strict hygiene protocols and disposable medical equipment, along with the routine implementation of universal precautions in medical, dental, and aesthetic settings have significantly reduced the risk of iatrogenic HCV transmission. A crucial role in the decrease of HCV prevalence has been played by the increasing availability of DAA since 2015 [2,3,4]. Likewise, data from Southern Italy demonstrated that screening approaches tailored to local epidemiology, even in the context of challenges such as the COVID-19 pandemic, can improve case detection and guide elimination strategies [5]. These findings underscore the evolving HCV landscape in Italy and highlight the need to adapt management strategies to regional dynamics.

In light of this changing epidemiology, it is important to recall how therapeutic approaches have evolved over time, with outstanding improvements achieved over the last few years in the care of HCV patients [4]. The advent of long-acting pegylated interferon ribavirin (Peg-IFN–RBV) represented one major cornerstone in HCV therapy and remained the standard of care for a decade (2001–2011) [4,5,6,7]. These combinations, administrated for 24–48 weeks of treatment, allowed for a sustained virological response (SVR) rate, ranging between 40% and 70%, but their use is associated with a high incidence of adverse effects and a significant decline in patients’ quality of life [8]. Since 2011, several direct-acting antiviral agents (DAAs) have been developed which targeted three proteins involved in different key steps of the HCV life cycle: NS3/4A protease, NS5A protein, and NS5B RNA-dependent RNA polymerase [9]. These drugs led to substantial improvements in the SVR rate (to over 90%), with shorter durations of treatment of 8, 12, 16, or 24 weeks and a very good safety profile, enabling access to treatment in nearly all HCV-infected patients [10,11].

The advent of two pangenotypic DAA combinations marked a further significant advancement in HCV treatment, since these regimens not only improved efficacy but also enabled treatment without the need for HCV genotyping and, in certain cases, without prior fibrosis assessment [12,13,14,15,16]. Phase II and phase III clinical trials have described good safety and efficacy profiles of pDAAs across various HCV genotypes [17,18]. Additionally, abundant real-world evidence has been accumulating and confirming the outcomes of the randomized controlled studies [19,20].

Nevertheless, none of the available DAAs is completely free of drug–drug interactions (DDIs), which have the potential to alter the pharmacokinetics of these agents, thereby influencing both their therapeutic efficacy and toxicity. Extensive studies on the interactions between DAAs and a range of critical co-administered medications have been conducted [21,22,23]. Since HCV causes important extrahepatic manifestations [24,25], patients frequently have multiple comorbidities requiring complex polypharmacy regimens, and an estimated 30% to 60% of those receiving DAA treatment may be at risk for clinically significant DDIs [26,27,28,29]. This scenario may be even more complicated in the elderly population that usually bears a multimorbid profile with an intrinsic higher risk of DDIs among concomitant medications [27]. The guidelines of the European Association for the Study of the Liver (EASL) indicate that the assessment of DDIs should be undertaken prior starting a DAA treatment, with full details on all prescribed therapies, over-the-counter (OTC) drugs, vitamin supplementation, and illicit drugs [30].

In Italy, two pDAAs, SOF/VEL and GLE/PIB, have been available since 2017 [31,32]. The intrinsic risk of DDIs is one differentiating factor between SOF/VEL and GLE/PIB. It has been extensively reported that GLE/PIB-associated DDI risk is higher compared to that of SOF/VEL because glecaprevir (GLE) is a protease inhibitor that can significantly alter the metabolism of other drugs, mainly by inhibiting cytochrome P450 enzymes (such as CYP3A4) [28,33,34].

In a nutshell, SOF/VEL and GLE/PIB are similar in terms of their SVR and safety profile, but the choice between each of the two therapeutic options should consider the patient’s characteristics, comorbidity profile, and co-medications; evaluation of the benefit/risk profile; and the patient’s preferences [29]. Understanding these characteristics is crucial for selecting the most appropriate therapy for HCV patients to maximize treatment benefits. Comparing the two regimens becomes even more essential in complex cases, such as patients with concomitant compensated cirrhosis, hard-to-treat genotypes, or those with comorbidities [35].

This real-world analysis was undertaken to describe HCV patients receiving SOF/VEL or GLE/PIB in Italy, focusing on the clinical, pharmacological, and epidemiological drivers for pDAA choice and on the potential gaps in the medical practice when prescribing the most suitable antiviral to each individual subject.

## 2. Materials and Methods

### 2.1. Data Source

A retrospective observational analysis was conducted using the administrative databases of Italian healthcare entities, covering around 3.7 million health-assisted citizens, and with data available from 2009 to 2023. For the current analysis, healthcare entities were selected by their geographical distribution (the north/centre/south of Italy), by data completeness, and by the high-quality linked datasets. The following databases were browsed: i) demographic database, to obtain all patient demographic data, such as sex, age, and date of death (if applicable); ii) pharmaceuticals database, to collect information on medicinal products reimbursed by the NHS as the Anatomical-Therapeutic Chemical (ATC) code, number of packages, number of units per package, unit cost per package, and prescription date; iii) hospitalization database, for all hospitalization data, including the discharge diagnosis codes classified according to the International Classification of Diseases, Ninth Revision, Clinical Modification (ICD-9-CM), Diagnosis-Related Group (DRG), and DRG-related charge (provided by the Health System); iv) outpatient specialist services database, to gather all information about visits and diagnostic tests (date and type of provision, type of activity, and laboratory test or specialist visit charge); v) payment exemption database, to collect data of the payment waiver codes by which patients are discharged from paying in the presence of certain specific diagnoses.

The dataset used consists solely of anonymized data. All the results of the analyses were produced and presented as aggregated summaries. Approval has been obtained from the ethics committees of the participating healthcare entities.

### 2.2. Study Design, Study Population, and Patient Cohorts’ Definition

All HCV adult patients prescribed with pDAAs, namely, SOF/VEL (ATC code J05AP55) or GLE/PIB (ATC code J05AP57), were included from 1 January 2018 to 31 December 2023 (inclusion period). The date of the first prescription for pDAAs during the inclusion period was defined as the index-date. The characterization period comprised all the time of data availability before the index-date (at least 12 months), and patients were followed-up from the index-date until the end of data availability (at least 12 months).

People aged < 18 years, or without continuous data availability during the study period (for instance those who moved to another region) or with missing data, were excluded from the analysis. The included patients were then divided into two mutually exclusive cohorts based on the type of pDAAs prescribed at index-date, namely, SOF/VEL cohort and GLE/PIB cohort.

### 2.3. Analysis of Demographic and Clinical Characteristics of Patients

For all the included patients, baseline demographic characteristics, in terms of age and sex distribution (expressed as percentage of males), were collected at index-date. The comorbidity profile was evaluated using the Charlson Comorbidity Index (CCI), a score resulting from the sum of 19 weighted concomitant diseases [36]. The clinical status was also assessed through the assessment of the following conditions during the characterization period: cardiovascular disease (CVD), diabetes, drug addiction, mental disorders, tumours, and cirrhosis. These diseases were detected in the databases using specific diagnosis proxies: hospitalizations and exemption codes were searched during all available periods before the index-date, while drug prescriptions and outpatient specialist services were searched during the year before the index-date.

The overall population of pDAA-treated patients stratified by treatment, GLE/PIB vs. SOF/VEL, was also investigated during the characterization period in terms of most frequent hospitalizations, type of active exemption codes, and treatment patterns. The prescriptions of all possible treatments, excluding pDAAs (ATC code J05AP), were collected and classified along with the first or second level of ATC code. The proportion of patients prescribed with a specific drug class was reported.

### 2.4. Statistical Analysis

Continuous variables were reported as mean ± standard deviation (SD); categorical variables were expressed as numbers and percentages. Statistical significance was accepted at *p* < 0.05. Comparative analyses were performed by Student’s *t* test for continuous variables and chi-square test for categorical variables, as appropriate. All analyses were performed using Stata SE version 12.0 (StataCorp, College Station, TX, USA).

## 3. Results

As Table 1 shows, among the 5565 pDAA-treated patients included, 49% received GLE/PIB and 51% received SOF/VEL. The GLE/PIB-treated patients were significantly younger than the SOF/VEL-treated patients (mean age 57.6 vs. 60.8 years, *p* < 0.001), with a median age of 56 years (range 18–94) for GLE/PIB and 58 years (range 19–96) for SOF/VEL.

The average CCI in the overall population of patients on pDAA therapy was 0.5, lower in the GLE/PIB cohort with respect to the SOF/VEL cohort (0.4 vs. 0.6, *p* < 0.001). The most frequent comorbidities were mental disorders (15.9%) and diabetes (12.3%). The patients receiving SOF/VEL showed a tendentially higher prevalence of all comorbidities, and the difference met statistical significance for diabetes (14.0% vs. 10.5%, *p* < 0.001), mental disorders (17.7% vs. 14.1%, *p* < 0.001), cancer (12.2% vs. 7.8%, *p* < 0.001), and cardiovascular/cerebrovascular diseases (15.7% vs. 8.1%, *p* < 0.001).

This milder clinical picture in the GLE/PIB cohort compared to the SOF/VEL cohort was corroborated by the patterns of the most frequent previous hospitalizations (Table 2A) and active exemption codes (Table 2B). SOF/VEL-treated patients required more frequent hospitalizations for complications of the hepatobiliary system and pancreas (16% vs. 10.3%, *p* < 0.001), circulatory system (9.6% vs. 8.1%, *p* < 0.050), and digestive system (9.2% vs. 7.5%, *p* < 0.050) compared to the GLE/PIB-treated cohort. Likewise, SOF/VEL-treated patients more commonly had active exemption codes relating to diagnoses for hypertension (11.8% vs. 8.5%, *p* < 0.001), diabetes (8.5% vs. 5.4%, *p* < 0.001), HIV infection (4.8% vs. 2.0%, *p* < 0.001), liver cirrhosis and biliary cirrhosis (4.7% vs. 1.3%, *p* < 0.001), and circulatory system diseases (2.8% vs. 1.8%, *p* < 0.010).

**Figure 1:**
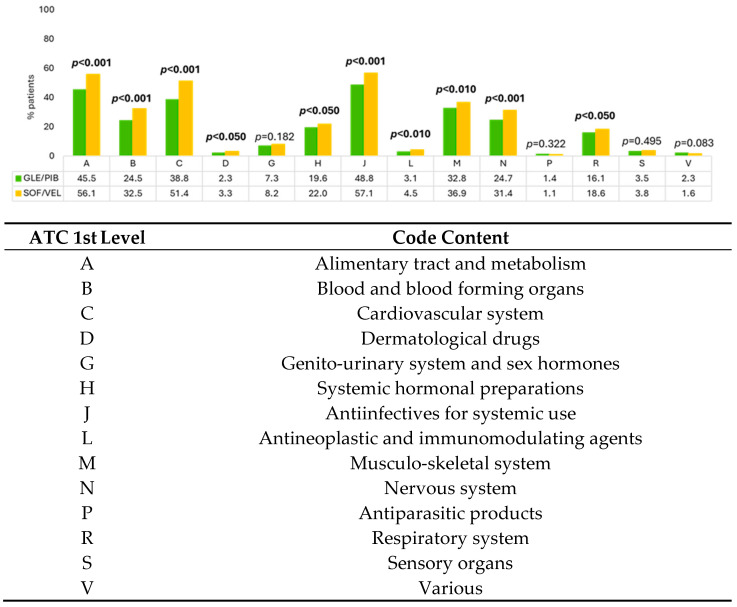
Presence of treatments classified by the first ATC level during characterization period in GLE/PIB-treated vs. SOF/VEL-treated patients. Significant *p* values are highlighted in bold.

The analysis of the prescription patterns revealed that 17% of the GLE/PIB-treated patients and 25% of the SOF/VEL-treated patients received ≥10 different prescriptions. As shown in Figure 1, most of the drugs classified by the first level of ATC code were more commonly prescribed in SOF/VEL-treated vs. GLE/PIB-treated patients, with the exception of codes G (genito-urinary system and sex hormones), P (antiparasitic products), S (sensory organs), and V (various).

The polypharmacy used during GLE/PIB-treated vs. SOF/VEL-treated patients was stratified according to therapeutic classes, especially to identify drugs most relevant for DDIs, as shown in Figure 1.

## 4. Discussion

This real-world analysis provided a description of Italian patients with HCV infection receiving SOF/VEL or GLE/PIB, focusing on clinical, pharmacological, and epidemiological factors influencing pDAA selection and the current clinical challenges in prescribing the most appropriate antiviral therapy. Although GLE/PIB and SOF/VEL are comparable in terms of their SVR and safety profiles, each of them is characterized by specific features to be kept in consideration in therapeutic decisions. The prescribing patterns observed in this analysis reflect a rational approach consistent with good clinical practice and in line with current recommendations from both the EASL [30] and the Italian Association for the Study of the Liver (AISF) [37]. Treatment choices appeared to be guided by careful consideration of patient characteristics, comorbidity profiles, and the potential for DDIs, which are key factors emphasized by international guidelines. Within this framework, SOF/VEL was preferentially prescribed for older individuals with more complex clinical profiles and extensive concomitant therapies, while GLE/PIB was more frequently used in patients with fewer coexisting conditions and simpler medication regimens. These patterns suggest an individualized approach to HCV therapy in routine practice, aligning therapeutic decisions with the clinical needs of different patient populations.

Beyond clinical profiles and DDI considerations, the involvement of patients in shared decision-making can improve treatment uptake and adherence. Recent evidence from Italian hospital and drug addiction services has highlighted that aligning treatment strategies with patient preferences enhances engagement and satisfaction, thereby facilitating SRV and supporting broader HCV elimination goals [38].

Since the development of the first DAAs, the majority of clinical trial participants were typically healthy individuals with minimal comorbidities and limited use of concomitant pharmacological treatments [39,40,41]. For this reason, real-word evidence might be valuable support to integrate clinical trials, because the data are generated from unselected populations mirroring the standard clinical practice [42,43,44,45,46]. We have shown the demographic and clinical features of the population included in the present analysis and confirmed that individuals with chronic HCV infection often present with clinical complexity, which is further exacerbated by aging and the presence of additional comorbidities requiring polypharmacy regimens [27]. The clinical implications of established or potential DDIs between DAAs and comedications can be variable, resulting in altered drug concentrations with either reduced efficacy or increased toxicity. Thus, assessing these interactions in HCV patients, especially those with a more severe clinical status, is important to increase the number of patients on treatment and treatment adherence. Frequently, patients with severe comorbidities fear to start another treatment, having complex concomitant treatments for chronic diseases. Since 2015, the PITER Cohort Study (Piattaforma Italiana per lo studio della Terapia delle Epatiti viRali) has provided an overview of variations in comorbidity profiles and potential DDIs among patients with chronic HCV infection enrolled and treated with DAA across more than 60 Italian centres. Patients showed high SVR rates with DAAs, resulting in improved liver function and fibrosis, with some reversal of liver damage, even though DDIs remained a critical concern [23]. SVR indeed is not traceable in the administrative databases used for our analysis because laboratory data are not collected. It is worth noting that the monitoring registries of the Italian Medicines Agency (AIFA), although essential for tracking treatment access and eligibility, do not collect laboratory outcomes such as SVR [46]. In this context, the PITER cohort plays a key role in filling this information gap, as it integrates clinical and laboratory data and has consistently reported SVR rates above 96% with interferon-free DAAs across different genotypes and disease stages, independently by the DAA regimen prescribed [23,47]. Taken together, these findings support the expectation that similarly high SVR rates would apply to the population described here. Nevertheless, although data from the PITER cohort confirmed that SVR rates were similarly high with both SOF/VEL and GLE/PIB regimens, consistent with randomized trials and other real-world evidence [23,48], clinical centres still carefully adhere to guideline recommendations and systematically assess potential DDIs, comorbidity profiles, and individual patient characteristics when selecting therapies [30,35,37,49]. This tailored approach in real-world practice is crucial to ensure that the excellent SVR outcomes observed in clinical trials are maintained in diverse and more complex patient populations [23,47].

The introduction of pDAAs, effective against all HCV genotypes, has simplified HCV treatment, as they eliminate the need for genotype-specific therapies and enable a more streamlined approach to managing the infection [48,49]. When choosing SOF/VEL and GLE/PIB for HCV treatment, DDIs have an important role. Patients on multiple concomitant medications, especially those requiring drugs with known interactions, may face increased risks of adverse events with protease inhibitors due to its higher DDI profile. The results of the present analysis are, in general, consistent with the view that the SOF/VEL is preferred as a more suitable option in populations with polypharmacy regimens, potentially to minimize potential DDIs [20,27,28,35,49,50,51,52]. In the pDAA-treated population included here, SOF/VEL-treated patients were older, had a worse comorbidity profile documented by a higher CCI, and were more commonly burdened by coexisting conditions like diabetes, mental disorders, cancer, and cardiovascular diseases. This difference was also reflected in hospitalization patterns, with SOF/VEL-treated patients more frequently hospitalized for complications related to the liver and pancreas. Additionally, SOF/VEL patients had higher rates of active exemption codes for chronic conditions like hepatitis, hypertension, and diabetes, suggesting a more severe clinical profile compared to that of the GLE/PIB group. Our findings are consistent with a retrospective study analysing data of 1718 pDAA-treated adults in the US electronic medical record database which reported DDIs in 41% of patients on GLE/PIB and 27% on SOF/VEL. Commonly interacting medications included statins, antipsychotics, calcium channel blockers, and proton pump inhibitors, similar to our analysis where cardiovascular drugs and drugs to treat nervous system conditions, the alimentary tract, and metabolism were more commonly prescribed in the SOF/VEL cohort than in the GLE/PIB cohort [28]. Clinical trials and real-world studies have consistently reported comparable safety and efficacy (SVR rates above 95%) for both SOF/VEL and GLE/PIB, even in challenging populations such as patients with cirrhosis or a history of prior treatment failure [53,54,55]. Even though GLE/PIB is approved for shorter treatment durations (8 weeks in many cases) compared to the standard 12 weeks of SOF/VEL, the latter offers lower potential for DDIs, which is particularly relevant in patients with multiple comorbidities or complex treatment regimens [26]. In certain subgroups, such as those with decompensated cirrhosis or significant renal impairment, tailored regimens or longer treatment courses may still be required.

Importantly, the main findings of the present analysis are in line with the latest Italian real-world data on pDAA treatments between 2017 and 2023 collected by the PITER Collaborating Group Real [56]. The PITER analysis of 1208 SOF/VEL-treated and 754 GLE/PIB-treated patients confirmed that those receiving the SOF/VEL option, associated with fewer potential DDIs, were 5 years older (considering the median age) and were more commonly burdened by ≥4 comorbidities compared to the GLE/PIB-treated population (respectively, 5.0% and 2.4%). The pattern of concomitant medications by category also mirrored that of this analysis as cardiovascular medications (i.e., antihypertensives, antithrombotics, and lipid-lowering therapies) and treatments related to the nervous and digestive systems were significantly more represented in the 1208 SOF/VEL-treated patients than in the GLE/PIB-treated patients [56].

Although cost analysis was beyond the scope of the present study and therefore not directly evaluated in our datasets, economic considerations are highly relevant in the context of HCV elimination strategies. Previous modelling in Italian cohorts has shown that investing in broad HCV strategies through early pDAA treatment, expanded screening, and universal treatment policies is highly cost-effective and leads to significant long-term savings and health gains for the Italian NHS [57,58,59]. A European study covering England, Italy, Romania, and Spain estimated that for every 1000 standardized patients treated with DAAs between 2015 and 2019, over 1200 cases of hepatocellular carcinoma, decompensated cirrhosis, and liver transplantation could be avoided in Italy, with associated cost savings ranging from EUR 45 to 275 million. In Italy, the investment in DAAs was projected to reach a break-even point in only 5.4 years, underlining the rapid return on investment and long-term economic benefits of early and widespread treatment access. A delay in treatment initiation, such as those seen during the COVID-19 pandemic, was projected to increase liver-related mortality, further reinforcing the importance of maintaining and expanding timely DAA access to sustain HCV elimination efforts [59]. The limitations of the present analysis are inherent to its observational design and reliance on administrative databases. These data sources are primarily intended for reimbursement tracking and may lack detailed clinical information on comorbidities, adverse events, and other potential confounders. Some conditions, such as overweight/obesity, steatosis, prior interferon use, or HCV genotype, could not be retrieved, and certain diagnoses may be underestimated due to the use of proxies like hospitalizations, exemption codes, and reimbursed drugs. Moreover, administrative databases do not capture patient preferences, adherence patterns, psychosocial context, or perceived treatment burden. Finally, laboratory outcomes such as SVR were not available.

## 5. Conclusions

The present analysis indicates that SOF/VEL was more frequently prescribed to older patients with more complex clinical profiles, characterized by a greater burden of comorbidities and a higher use of concomitant medications, likely reflecting its more favourable DDI profile. Conversely, GLE/PIB was more commonly prescribed in patients with fewer comorbidities and less extensive polypharmacy. Although both regimens are recognized in current practice as effective and safe therapeutic options, their use should be guided by the specific characteristics of the populations to which they are prescribed. These findings, generated from the real clinical practice in Italy, underscore the importance of aligning the selection of pDAAs with individual patient clinical profiles in order to support appropriate treatment decisions and ensure optimal use of available therapeutic strategies in HCV care.

## Figures and Tables

**Table 1:** Baseline demographic and clinical characteristics of overall pDAA-treated patients, GLE/PIB-treated patients, and SOF/VEL-treated patients. Comparisons were performed between GLE/PIB-treated vs. SOF/VEL-treated patients, and significant *p* values are highlighted in bold.

	Overall	GLE/PIB-Treated	SOF/VEL-Treated	*p*
N. of patients, N (%)	5565	2735 (49%)	2830 (51%)	
Male sex, N (%)	3384 (60.8%)	1641 (60.0%)	1743 (61.6%)	0.224
Age, years, mean ± SD	59.2 ± 15.0	57.6 ± 14.9	60.8 ± 15.0	**<0.001**
18–35 years old, N (%)	304 (5.5%)	150 (6.6%)	156 (4.4%)	**<0.001**
36–55 years old, N (%)	2188 (39.3%)	1075 (42.3%)	1112 (36.5%)	
≥56 years old, N (%)	3073 (55.2%)	1510 (51.2%)	1562 (59.2%)	
CCI, mean ± SD	0.5 ± 1.0	0.4 ± 0.9	0.6 ± 1.1	**<0.001**
CCI = 0	3964 (71.2%)	1947 (74.9%)	2015 (67.7%)	**<0.001**
CCI = 1–2	1248 (22.4%)	613 (20.3%)	634 (24.5%)	
CCI = 3–4	301 (5.4%)	148 (4.0%)	153 (6.8%)	
CCI ≥ 5	52 (0.9%)	25 (0.9%)	25 (1.0%)	
CVD	398 (7.2%)	183 (6.7%)	215 (7.6%)	0.157
Diabetes	683 (12.3%)	287 (10.5%)	396 (14.0%)	**<0.001**
Drug addiction	117 (2.1%)	52 (1.9%)	65 (2.3%)	0.224
Mental disorders	886 (15.9%)	386 (14.1%)	501 (17.7%)	**<0.001**
Tumors	558 (10.0%)	213 (7.8%)	345 (12.2%)	**<0.001**
Cirrhosis	665 (11.9%)	222 (8.1%)	444 (15.7%)	**<0.001**

Abbreviations: CCI, Charlson Comorbidity Index; CVD, cardiovascular disease; GLE/PIB, glecaprevir/pibrentasvir; SD, standard deviation; SOF/VEL, sofosbuvir/velpatasvir.

**Table 2:** (**A**) Most frequent previous hospitalizations and (**B**) active exemption codes among overall pDAA-treated patients, GLE/PIB-treated patients, and SOF/VEL-treated patients. Comparisons were performed in GLE/PIB-treated vs. SOF/VEL-treated patients, and significant *p* values are highlighted in bold.

	Overall (N = 5565)	GLE/PIB-Treated (N = 2735)	SOF/VEL-Treated (N = 2830)	*p*
**A. Hospitalizations, N (%)**				
Hepatobiliary system and pancreas	735 (13.2%)	282 (10.3%)	453 (16.0%)	**<0.001**
Musculoskeletal system and connective tissue	695 (12.5%)	337 (12.3%)	358 (12.7%)	0.711
Circulatory system	495 (8.9%)	222 (8.1%)	273 (9.6%)	**<0.050**
Digestive system	465 (8.4%)	205 (7.5%)	260 (9.2%)	**<0.050**
Respiratory system	332 (6.0%)	156 (5.7%)	176 (6.2%)	0.658
Nervous system	259 (4.7%)	115 (4.2%)	144 (5.1%)	0.118
Skin, subcutaneous tissue, and breast	255 (4.6%)	122 (4.5%)	133 (4.7%)	0.670
Kidney and urinary tract	226 (4.1%)	142 (5.2%)	- *	-
Ear, nose, mouth, and throat	194 (3.5%)	94 (3.4%)	100 (3.5%)	0.844
Mental diseases and disorders	167 (3.0%)	- *	116 (4.1%)	-
**B. Exemption codes, N (%)**				
Chronic hepatitis	2151 (38.7%)	1102 (40.3%)	1049 (37.1%)	**<0.050**
Hypertension	566 (10.1%)	234 (8.5%)	332 (11.8%)	**<0.001**
Diabetes mellitus	390 (7.0%)	149 (5.4%)	241 (8.5%)	**<0.001**
Malignant neoplastic diseases	377 (6.8%)	153 (5.6%)	224 (7.9%)	**<0.001**
Dependence on drug, psychotropic, and alcohol substances	363 (6.5%)	174 (6.4%)	189 (6.7%)	0.633
HIV infection	192 (3.5%)	56 (2.0%)	136 (4.8%)	**<0.001**
Liver cirrhosis, biliary cirrhosis	169 (3.0%)	36 (1.3%)	133 (4.7%)	**<0.001**
Circulatory system diseases	128 (2.3%)	48 (1.8%)	80 (2.8%)	**<0.010**
Congenital hypothyroidism, severe acquired hypothyroidism	70 (1.3%)	35 (1.3%)	35 (1.2%)	0.886
Chronic renal failure	52 (0.9%)	41 (1.5%)	- **	-

* Not in the top 10 most frequent hospitalizations; ** Not in the top 10 most frequent exemptions. Abbreviations: HIV, human immunodeficiency virus; GLE/PIB, glecaprevir/pibrentasvir; SOF/VEL, sofosbuvir/velpatasvir.

## Data Availability

The data supporting the findings of this article are available at an aggregated level from the authors upon reasonable request and with permission of the participating healthcare entities. Requests to access should be directed to the corresponding author.

## Abbreviations

The following abbreviations are used in this manuscript:

AISF	Italian Association for the Study of the Liver
ATC	Anatomical Therapeutic Chemical
CCI	Charlson Comorbidity Index
CYP	Cytochrome P450
DAAs	Direct-Acting Antivirals
DDIs	Drug–Drug Interactions
DRG	Diagnosis-Related Group
EASL	European Association for the Study of the Liver
GLE	Glecaprevir
GLE/PIB	Glecaprevir/Pibrentasvir
HCV	Hepatitis C Virus
HIV	Human Immunodeficiency Virus
ICD-9-CM	International Classification of Diseases, Ninth Revision, Clinical Modification
NHS	National Health Service
NS3/4A	Nonstructural protein 3/4A (HCV protease)
NS5A	Nonstructural protein 5A (HCV replication complex)
NS5B	Nonstructural protein 5B (RNA-dependent RNA polymerase)
OTC	Over-the-Counter
Peg-IFN–RBV	Pegylated Interferon Ribavirin
pDAAs	Pangenotypic Direct-Acting Antivirals
PIB	Pibrentasvir
SD	Standard Deviation
SOF	Sofosbuvir
SOF/VEL	Sofosbuvir/Velpatasvir
SVR	Sustained Virological Response
WHO	World Health Organization
